# Two Years with a Tubeless Automated Insulin Delivery System: A Single-Arm Multicenter Trial in Children, Adolescents, and Adults with Type 1 Diabetes

**DOI:** 10.1089/dia.2023.0364

**Published:** 2024-01-05

**Authors:** Amy B. Criego, Anders L. Carlson, Sue A. Brown, Gregory P. Forlenza, Bruce W. Bode, Carol J. Levy, David W. Hansen, Irl B. Hirsch, Richard M. Bergenstal, Jennifer L. Sherr, Sanjeev N. Mehta, Lori M. Laffel, Viral N. Shah, Anuj Bhargava, Ruth S. Weinstock, Sarah A. MacLeish, Daniel J. DeSalvo, Thomas C. Jones, Grazia Aleppo, Bruce A. Buckingham, Trang T. Ly

**Affiliations:** ^1^ Department of Pediatric Endocrinology, International Diabetes Center, Park Nicollet, Minneapolis, Minnesota, USA.; ^2^ International Diabetes Center, Park Nicollet, HealthPartners, Minneapolis, Minnesota, USA.; ^3^ Division of Endocrinology, Center for Diabetes Technology, University of Virginia, Charlottesville, Virginia, USA.; ^4^ Department of Pediatrics, Barbara Davis Center for Diabetes, University of Colorado Anschutz Medical Campus, Aurora, Colorado, USA.; ^5^ Atlanta Diabetes Associates, Atlanta, Georgia, USA.; ^6^ Division of Endocrinology, Diabetes, and Metabolism, Icahn School of Medicine at Mount Sinai, New York, New York, USA.; ^7^ Department of Pediatrics, SUNY Upstate Medical University, Syracuse, New York, USA.; ^8^ Department of Medicine, University of Washington, Seattle, Washington, USA.; ^9^ Department of Pediatrics, Yale School of Medicine, New Haven, Connecticut, USA.; ^10^ Joslin Diabetes Center, Harvard Medical School, Boston, Massachusetts, USA.; ^11^ Iowa Diabetes Research, West Des Moines, Iowa, USA.; ^12^ Department of Medicine, SUNY Upstate Medical University, Syracuse, New York, USA.; ^13^ Department of Pediatrics, University Hospitals Cleveland Medical Center, Rainbow Babies and Children's Hospital, Cleveland, Ohio, USA.; ^14^ Department of Pediatrics, Baylor College of Medicine, Houston, Texas, USA.; ^15^ Department of Research, East Coast Institute for Research at The Jones Center, Macon, Georgia, USA.; ^16^ Division of Endocrinology, Metabolism, and Molecular Medicine, Feinberg School of Medicine, Northwestern University, Chicago, Illinois, USA.; ^17^ Division of Pediatric Endocrinology, Department of Pediatrics, Stanford University, Stanford, California, USA.; ^18^ Insulet Corporation, Acton, Massachusetts, USA.

**Keywords:** Type 1 diabetes, Artificial pancreas, Insulin pumps, Clinical trials, Closed-loop systems

## Abstract

**Background:**

The Omnipod^®^ 5 Automated Insulin Delivery (AID) System was shown to be safe and effective following 3 months of use in people with type 1 diabetes (T1D); however, data on the durability of these results are limited. This study evaluated the long-term safety and effectiveness of Omnipod 5 use in people with T1D during up to 2 years of use.

**Materials and Methods:**

After a 3-month single-arm, multicenter, pivotal trial in children (6–13.9 years) and adolescents/adults (14–70 years), participants could continue system use in an extension phase. HbA1c was measured every 3 months for up to 15 months; continuous glucose monitor metrics were collected for up to 2 years.

**Results:**

Participants (*N* = 224) completed median (interquartile range) 22.3 (21.7, 22.7) months of AID. HbA1c was reduced in the pivotal trial from 7.7% ± 0.9% in children and 7.2% ± 0.9% in adolescents/adults to 7.0% ± 0.6% and 6.8% ± 0.7%, respectively, (*P* < 0.0001), and was maintained at 7.2% ± 0.7% and 6.9% ± 0.6% after 15 months (*P* < 0.0001 from baseline). Time in target range (70–180 mg/dL) increased from 52.4% ± 15.6% in children and 63.6% ± 16.5% in adolescents/adults at baseline to 67.9% ± 8.0% and 73.8% ± 10.8%, respectively, during the pivotal trial (*P* < 0.0001) and was maintained at 65.9% ± 8.9% and 72.9% ± 11.3% during the extension (*P* < 0.0001 from baseline). One episode of diabetic ketoacidosis and seven episodes of severe hypoglycemia occurred during the extension. Children and adolescents/adults spent median 96.1% and 96.3% of time in Automated Mode, respectively.

**Conclusion:**

Our study supports that long-term use of the Omnipod 5 AID System can safely maintain improvements in glycemic outcomes for up to 2 years of use in people with T1D.

**Clinical Trials Registration Number:**

NCT04196140

## Introduction

Many people with type 1 diabetes (T1D) are not meeting recommended glycemic targets across all ages and demographic populations, contributing to the immense burden on those living with diabetes and their families.^1–5^ The relatively recent introduction of automated insulin delivery (AID) systems has shown promising results for improving glycemic outcomes for people with diabetes, with several systems now available in the United States and Europe.^6–10^

The Omnipod^®^ 5 AID System (Insulet Corporation, Acton, MA) is the first wearable tubeless AID system cleared for use in people with T1D aged 2 years and older. It consists of an on-body insulin-filled pump with a built-in algorithm (Pod) and the Omnipod 5 App as a controller and is interoperable with a continuous glucose monitor (Dexcom G6; Dexcom, San Diego).

The Omnipod 5 System has been evaluated in a 3-month single-arm, multicenter pivotal clinical trial that demonstrated effectiveness and safety, with an improvement of 15.6% ± 11.5% in time in range (TIR) 70–180 mg/dL for children and 9.3% ± 11.8% for adolescents and adults when using Omnipod 5 compared with standard therapy.^11^ Omnipod 5 use also resulted in significant reductions in HbA1c and hyperglycemia across all participant age groups, with reductions in hypoglycemia also reported in adolescents and adults. Further, the observed incidence rates for severe hypoglycemia and diabetic ketoacidosis were low during the pivotal trial (4.8 and 1.2 events per 100 person-years, respectively).

To evaluate the durability of these glycemic benefits, participants were offered the ability to continue using the Omnipod 5 System in an optional extension phase of the trial. The objective of this study was to evaluate the long-term safety and effectiveness of the Omnipod 5 AID System in children, adolescents, and adults with T1D during up to 2 years of at-home use.

## Materials and Methods

This study is an extension of a 3-month outpatient pivotal clinical trial of the Omnipod 5 AID System (Insulet Corporation, Acton, MA). Full details on the study design for the pivotal trial have been published previously.^11^ In brief, the pivotal trial included 240 participants (112 children aged 6–13.9 years and 128 adolescents and adults aged 14–70 years) with T1D for at least 6 months with an HbA1c < 10% (86 mmol/mol) at screening (complete eligibility criteria in Supplementary Table S1). The participants completed a 2-week standard therapy phase using their usual insulin regimen followed by 3 months of AID with the Omnipod 5 System. Participants from the pivotal trial could then opt to continue using the system as part of the extension phase described here.

### Study conduct and oversight

A central Institutional Review Board and local review boards approved the protocol for this study. Informed consent was obtained from adults (18 years and older), and assent and consent for participants younger than 18 years were obtained from their parents and guardians, according to state requirements. The United States Food and Drug Administration approved an investigational device exemption for use in the pivotal trial and extension phase. A Medical Monitor and independent Data and Safety Monitoring Board provided oversight. The trial was registered at clinicaltrials.gov.

### Study design and participants

Participants completing the pivotal trial were offered the opportunity to continue with the optional extension phase, which began between July 10, 2020 and September 18, 2020. The Omnipod 5 System was cleared for commercial use by the FDA on January 27, 2022, at which point participants were transitioned out of the study with the last participant visit on April 20, 2022. This resulted in an extension phase duration of median (interquartile range [IQR]) 19.2 (18.6, 19.7) months for a total use of 22.3 (21.7, 22.7) months, with a maximum of 23.5 months (inclusive of the 3-month pivotal study).

There were 10 follow-up visits during the extension phase of the study occurring every 30 days (first 6 visits) or every 45 days (next 4 visits), corresponding to a total of 15 months of AID system use, after which visits were continued every 60 days (Supplementary Table S2). Each visit included a review of device data, which was automatically and continuously uploaded throughout the trial, and participants were asked to report on medication use, adverse events, and device issues. Adjustments in system settings were made as needed at each visit. HbA1c was measured every 3 months ending at 15 months of total Omnipod 5 System use, while glucose sensor and device data were collected over the entire extension period (up to 2 years total use).

### Investigational device

The investigational device consisted of a tubeless on-body insulin pump (Pod), with an embedded AID algorithm, interoperable on-body glucose sensor, and a mobile application on a locked-down Android phone. The AID algorithm enables the delivery of microboluses of insulin every 5 min based on current and projected glucose values to bring blood glucose levels toward the user-selected target (between 110 and 150 mg/dL in 10 mg/dL increments, customizable by time of day). The app was used to calculate and deliver user-initiated meal and correction boluses. Additional details on the device components and use have been published previously.^12^

### Outcomes

Primary effectiveness outcomes were differences in HbA1c during the extension phase compared to baseline (before Omnipod 5) and percentage of time in glucose target range (70–180 mg/dL, “TIR”) during the extension phase compared to the standard therapy phase. Primary safety outcomes were incidence rates of severe hypoglycemia and diabetic ketoacidosis. Secondary outcomes included differences between the extension phase and the standard therapy phase for glucose metrics collected via glucose sensor (e.g., percent of time <54, <70, >180, ≥250, ≥300 mg/dL; and mean, standard deviation [SD], coefficient of variation of sensor glucose), clinical measures (e.g., total daily dose of insulin, total daily basal or bolus insulin delivery, body mass index [BMI]), and system use measures (e.g., time spent in Automated Mode, device deficiencies).

### Statistical methods

Analyses were performed using a modified intention-to-treat dataset of participants who entered the extension phase. Continuous variables were summarized using descriptive statistics. Categorical variables were summarized by frequencies and percentages. Comparisons were analyzed using paired *t*-tests, or by Wilcoxon signed-rank tests if the Shapiro-Wilk tests of normality were significant (*P* < 0.05) or there were fewer than 10 participants in a group. No imputations of missing data were performed. Analyses were conducted separately for children and for adolescents and adults. All *P*-values were considered significant at a two-sided significance level of 5%. Analysis was performed using SAS version 9.4.

## Results

### Participants

Between July 10, 2020 and September 18, 2020, most participants (95%, 224/235) elected to continue into the optional extension phase following completion of the 3-month pivotal trial. Baseline characteristics of these 110 children and 114 adolescents and adults are detailed in Table 1. Ninety-one percent (204/224) of participants (98% [108/110] of children and 84% [96/114] of adolescents and adults) remained in the extension phase until the system became commercially available, with 86.2% of participants using the system for over 21 months. Participants who chose not to remain in the extension phase withdrew due to plans to conceive (*n* = 3), unrelated medical issues (*n* = 3), desire to use alternative glucose management (*n* = 8), or a general desire not to continue the study further (*n* = 6).

**Table 1.** Characteristics at Baseline for Those Electing to Participate in the Extension Phase

Characteristic	Children (6–13.9 years)	Adults (14–70 years)
*N*	110	114
Age (years)	10.4 ± 2.1 (6.0, 14.0^a^)	36.8 ± 14.0 (14.5, 69.8)
Duration of diabetes (years)	4.7 ± 2.6 (0.6, 11.6)	17.4 ± 11.4 (1.0, 49.7)
Body-mass index^b^	18.7 ± 3.2 (13.7, 32.4)	26.6 ± 4.8 (18.9, 41.4)
Female sex, no. (%)	59 (53.6)	68 (59.6)
Race/Ethnicity, no. (%)^c^		
White	102 (92.7)	103 (90.4)
Hispanic or Latino	8 (7.3)	4 (3.5)
Not Hispanic or Latino	94 (85.5)	99 (86.8)
Black or African American, White	3 (2.7)	—
Black or African American	2 (1.8)	5 (4.4)
Hispanic or Latino	—	1 (0.9)
Not Hispanic or Latino	2 (1.8)	4 (3.5)
Asian	—	2 (1.8)
Asian, White	2 (1.8)	—
Asian, Native Hawaiian or Pacific Islander, White	1 (0.9)	—
American Indian or Alaska Native, White	—	1 (0.9)
American Indian or Alaska Native	—	3 (2.6)
Hispanic or Latino	—	3 (2.6)
Not Hispanic or Latino	—	—
HbA1c (%)^d^	7.7 ± 0.9 (5.8, 10.3)	7.2 ± 0.9 (5.2, 9.8)
HbA1c (mmol/mol)^d^	61 ± 9.8 (40, 89)	55 ± 9.8 (33, 84)
Daily insulin dose (U/kg)^e^	0.85 ± 0.24 (0.25, 1.47)	0.61 ± 0.22 (0.19, 1.31)
Previous^f^ or current continuous glucose monitor use, no. (%)	106 (96.4)	112 (98.2)
Previous^f^ or current pump use, no. (%)	98 (89.1)	101 (88.6)
Using multiple daily injections as standard therapy method, no. (%)	13 (11.8)	19 (16.7)

Data are mean ± SD. Unless otherwise indicated, remaining values are range (minimum, maximum).

^a^ Age was determined at the date of informed consent. The birth date of one participant fell immediately after the informed consent date, resulting in their inclusion in the children cohort despite their age of 14.0 years after rounding.

^b^ Body-mass index is the weight in kilograms divided by the square of the height in meters.

^c^ Race and ethnicity were reported by the participants and are displayed exactly as reported. As shown, several participants chose more than one racial category. Ethnicity delineation is shown for racial categories where at least one person identified as Hispanic or Latino.

^d^ Participant eligibility for the study was determined using a point-of-care HbA1c measurement performed at screening, which in some cases differed from the laboratory assessment displayed here and used for analysis.

^e^ Baseline total daily insulin dose was determined from data collected during the standard therapy phase.

^f^ Previous use is defined as having used the device for any duration in the past.

SD, standard deviation.

### Glycemic outcomes

**FIG. 1.**
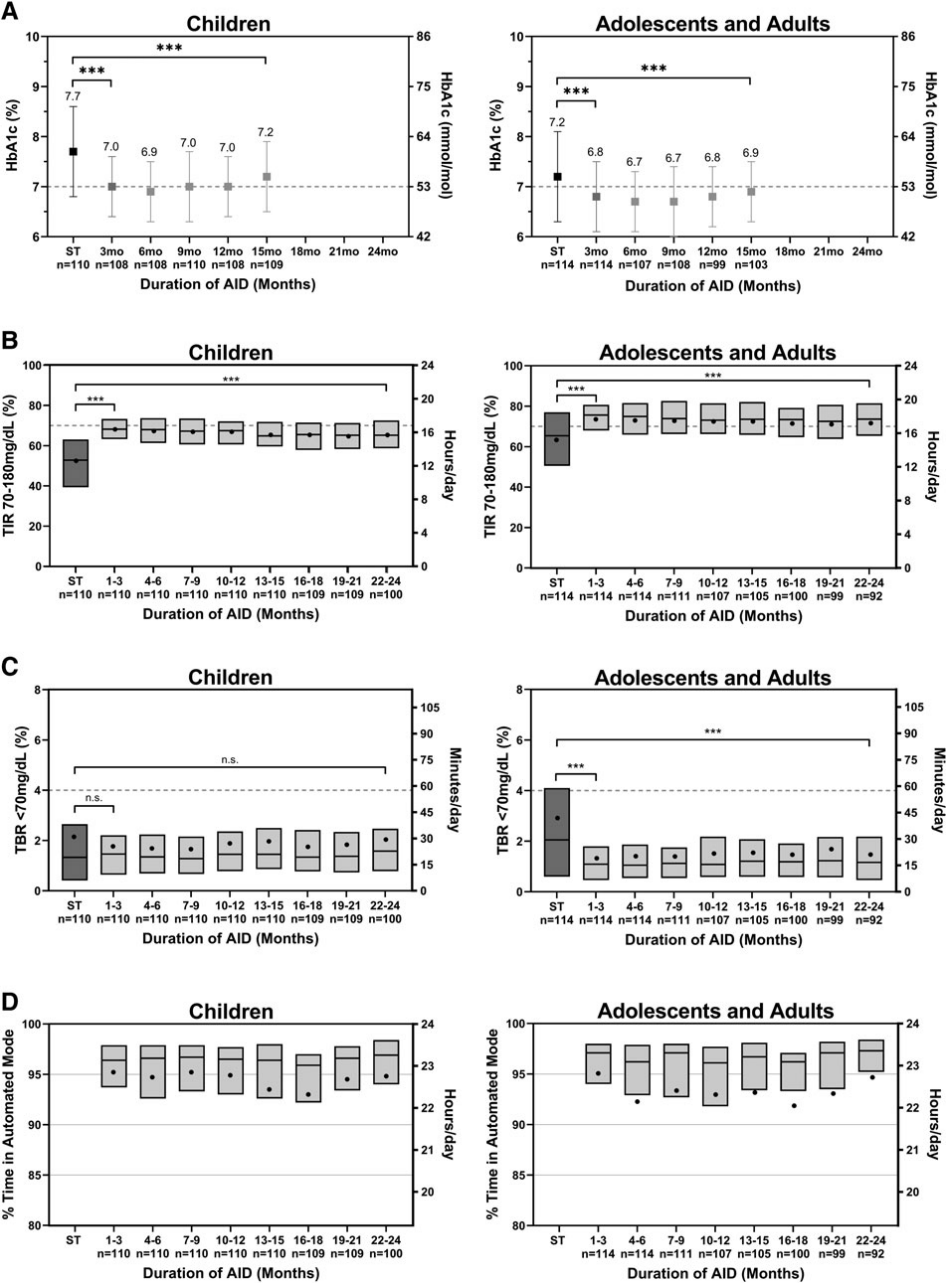
Glycemic outcomes and system use patterns during the ST phase, pivotal AID phase, and extension AID phase in 3-month intervals. **(A)** Mean HbA1c, **(B)** percentage time in target range (time in range) 70–180 mg/dL, **(C)** percentage time below range (<70 mg/dL), and **(D)** percentage of time in automated mode for children (age 6–13.9 years) (left) and adolescents and adults (age 14–70 years) (right) in 3-month intervals. **(A)** Error bars show the SD. **(B–D)** Box plots represent the median (line) with mean (dots). ***P < 0.001, n.s., not significant with *P* ≥ 0.05. AID, automated insulin delivery; SD, standard deviation; ST, standard therapy; TBR, time below range; TIR, time in range.

From baseline to 15 months of AID use, HbA1c was reduced by 0.5% ± 0.7% (5.5 ± 7.7 mmol/mol, *P* < 0.0001) in children, from 7.7% ± 0.9% (61 ± 9.8 mmol/mol) to 7.2% ± 0.7% (55 ± 7.7 mmol/mol), and reduced in adolescents and adults by 0.3% ± 0.6% (3.3 ± 6.6 mmol/mol, *P* < 0.0001), from 7.2% ± 0.9% (55 ± 9.8 mmol/mol) to 6.9% ± 0.6% (52 ± 6.6 mmol/mol) (Fig. 1A and Supplementary Table S3). Improvement was seen in both age groups regardless of baseline HbA1c, with those in the cohort with baseline HbA1c ≥ 8% (64 mmol/mol) maintaining a durable 1.0% (11 mmol/mol) improvement after 15 months (*P* < 0.0001) (Supplementary Fig. S1). The percentage of participants achieving the consensus target of HbA1c < 7.0% (53 mmol/mol) increased in both children (from 23% to 37%) and adolescents and adults (from 42% to 59%) from baseline to 15 months of AID use (Supplementary Table S4).

**FIG. 2.**
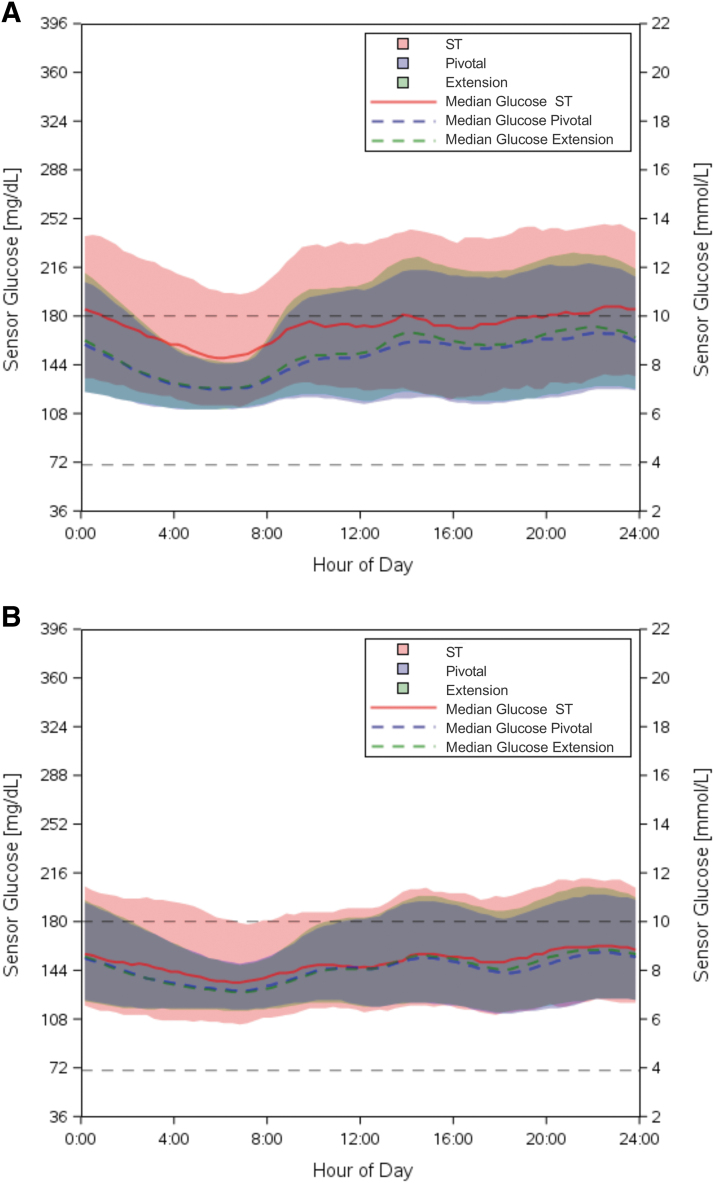
Median sensor glucose measurement for **(A)** children (age 6–13.9 years) and **(B)** adolescents and adults (age 14–70 years) during the ST phase (red solid line), pivotal AID phase (blue dashed line), and extension AID phase (green dashed line), with shaded area indicating the interquartile range for each phase. The target range (70–180 mg/dL) is indicated by the black dashed lines. Measurements represent a 24 h period from midnight to midnight. AID, automated insulin delivery; ST, standard therapy.

From standard therapy through the extension phase, TIR increased by 13.5% ± 12.0% in children (52.4% ± 15.6% to 65.9% ± 8.9%, *P* < 0.0001) and by 9.2% ± 11.6% in adolescents and adults (63.6% ± 16.5% to 72.9% ± 11.3%, *P* < 0.0001) (Tables 2 and 3), corresponding to an increase of 3.2 and 2.2 h/day, respectively. The improvement in TIR was stable over time when measured in 3-month intervals up to 24 months, as displayed in Figure 1B. Mean sensor glucose was also significantly reduced in both age groups (Tables 2 and 3). The improvements observed in these outcomes were largely maintained from the original 3-month pivotal study, with only minor decreases in TIR in both age groups (−2.0% ± 5.1% [*P* < 0.0001] in children and −0.9% ± 5.4% [*P* = 0.0411] in adolescents and adults) and a minor increase in mean glucose in children (4 ± 10 mg/dL [*P* < 0.0001]) from the pivotal phase to the extension phase. Sensor glucose profile by time of day for the standard therapy, pivotal, and extension phases is presented in Figure 2. The number of participants meeting established clinical targets^1^ for glycemic measures is included in Supplementary Table S4.

**Table 2.** Extension Study Results for Children (Ages 6–13.9 Years)

Parameter	Standard therapy phase	Pivotal study	Extension study	Change from ST to pivotal study	P-value^a^ ST versus pivotal	Change from ST to extension study	P-value^a^ ST versus extension	Change from pivotal to extension	P-value^a^ pivotal versus extension
*N*	110	110	110	110		110		110	
Phase duration	14 days	3 months	19.2 ± 1.0 months						
Percentage time 70–180 mg/dL, %	52.4 ± 15.6, 52.8 (39.3, 63.1)	67.9 ± 8.0, 68.2 (63.3, 73.3)	65.9 ± 8.9, 66.1 (60.0, 72.4)	15.5 ± 11.6, 15.0 (7.9, 24.6)	<0.0001^†^	13.5 ± 12.0, 12.8 (5.6, 20.6)	<0.0001^†^	−2.0 ± 5.1, −1.7 (−5.2, 1.2)	<0.0001^†^
Mean sensor glucose, mg/dL	184 ± 31, 181 (163, 205)	161 ± 15, 159 (150, 171)	164 ± 18, 165 (153, 176)	−23 ± 23, −21 (−36, −8)	<0.0001^††^	−19 ± 24, −16 (−32, −3)	<0.0001^††^	4 ± 10, 3 (−3, 10)	<0.0001^††^
SD of sensor glucose, mg/dL	68 ± 13, 68 (59, 77)	60 ± 9, 59 (52, 65)	63 ± 12, 62 (54, 69)	−9 ± 8, −8 (−14, −3)	<0.0001^†^	−6 ± 9, −6 (−12, 0)	<0.0001^†^	3 ± 6, 3 (−2, 6)	<0.0001^†^
Coefficient of variation of sensor glucose, %^b^	37.4 ± 5.1, 36.9 (33.7, 40.9)	37.0 ± 3.9, 37.6 (34.3, 39.8)	37.8 ± 4.1, 37.5 (35.4, 40.5)	−0.3 ± 4.2, −0.6 (−3.5, 2.5)	0.4060^†^	0.5 ± 4.4,0.4 (−2.6, 3.3)	0.2673^†^	0.8 ± 2.1, 0.7 (−0.6, 2.2)	0.0001^†^
Percentage time in glucose range, %									
<54 mg/dL	0.40 ± 0.83, 0.10 (0.00, 0.38)	0.32 ± 0.33, 0.22 (0.07, 0.41)	0.37 ± 0.39, 0.27 (0.11, 0.44)	−0.08 ± 0.67, 0.04 (−0.08, 0.21)	0.0551^††^	−0.03 ± 0.66, 0.07 (−0.06, 0.26)	0.0049^††^	0.05 ± 0.20, 0.03 (−0.05, 0.12)	0.0223^††^
<70 mg/dL	2.15 ± 2.63, 1.33 (0.41, 2.65)	1.77 ± 1.38, 1.46 (0.64, 2.21)	1.80 ± 1.40, 1.55 (0.75, 2.22)	−0.38 ± 1.97, 0.10 (−0.65, 0.69)	0.9752^††^	−0.35 ± 2.12, 0.14 (−0.86, 0.83)	0.9186^††^	0.04 ± 0.71, −0.02 (−0.25, 0.28)	0.8975^††^
>180 mg/dL	45.5 ± 16.6, 44.8 (35.3, 59.2)	30.3 ± 8.6, 29.7 (24.2, 35.5)	32.3 ± 9.5, 32.5 (25.7, 38.8)	−15.2 ± 12.2, −14.3 (−24.2, −7.0)	<0.0001^†^	−13.1 ± 12.7, −12.0 (−21.3, −4.8)	<0.0001^†^	2.0 ± 5.4, 1.9 (−1.5, 5.3)	0.0002^†^
≥250 mg/dL	19.1 ± 13.0, 15.9 (10.2, 26.3)	9.7 ± 5.4, 8.9 (5.7, 12.5)	11.3 ± 6.7, 10.3 (6.0, 15.3)	−9.4 ± 9.8, −6.1 (−14.1, −2.1)	<0.0001^††^	−7.8 ± 10.1, −5.2 (−12.8, −1.2)	<0.0001^††^	1.6 ± 3.9, 0.8 (−0.5, 3.7)	<0.0001^††^
≥300 mg/dL	8.5 ± 8.9, 5.8 (2.5, 11.0)	3.5 ± 2.9, 2.7 (1.4, 4.7)	4.6 ± 4.2, 3.4 (1.5, 6.1)	−5.0 ± 7.1, −2.6 (−7.5, −0.4)	<0.0001^††^	−4.0 ± 7.1, −2.3 (−6.1, 0.3)	<0.0001^††^	1.1 ± 2.7, 0.5 (−0.3, 2.1)	<0.0001^††^

Data are mean ± SD, median (IQR). To convert the values for glucose to millimoles per liter, multiply by 0.05551.

^a^ *P*-value determined using ^†^unadjusted two-sided paired *t*-tests or ^††^two-sided Wilcoxon signed rank test.

^b^ Coefficient of variation of sensor glucose is SD divided by the mean.

IQR, interquartile range; SD, standard deviation; ST, standard therapy.

**Table 3.** Extension Study Results for Adolescents and Adults (Ages 14–70 Years)

Parameter	Standard therapy phase	Pivotal study	Extension study	Change from ST to pivotal study	P-value^a^ ST versus pivotal	Change from ST to extension study	P-value^a^ ST versus extension	Change from pivotal to extension	P-value^a^ pivotal versus extension
*N*	114	114	114	114		114		114	
Phase duration	14 days	3 months	17.6 ± 4.2 months						
Percentage time 70–180 mg/dL, %	63.6 ± 16.5, 65.4 (50.5, 77.0)	73.8 ± 10.8, 75.6 (68.0, 80.7)	72.9 ± 11.3, 73.7 (66.2, 81.4)	10.2 ± 11.9, 8.7 (2.4, 17.0)	<0.0001^††^	9.2 ± 11.6, 8.1 (1.4, 16.6)	<0.0001^††^	−0.9 ± 5.4, −0.8 (−3.5, 1.8)	0.0411^††^
Mean sensor glucose, mg/dL	163 ± 28, 158 (139, 180)	154 ± 16, 151 (144, 163)	155 ± 18, 152 (143, 164)	−9 ± 20, −7 (−18, 6)	<0.0001^††^	−8 ± 20, −6 (−19, 5)	<0.0001^††^	1 ± 9, 1 (−4, 4)	0.2690^††^
SD of sensor glucose, mg/dL	58 ± 14, 56 (48, 67)	49 ± 11, 48 (42, 55)	51 ± 12, 51 (42, 57)	−8 ± 9, −8 (−14, −2)	<0.0001^†^	−7 ± 9, −6 (−12, 1)	<0.0001^††^	2 ± 5, 1 (−1, 4)	0.0011^††^
Coefficient of variation of sensor glucose, %^‡^	35.3 ± 5.9, 35.1 (30.6, 38.6)	31.8 ± 4.8, 32.2 (28.5, 34.8)	32.6 ± 5.0, 32.6 (29.8, 35.6)	−3.5 ± 4.5, −3.7 (−6.0, 0.0)	<0.0001^†^	−2.7 ± 4.9, −2.0 (−5.7, 0.7)	<0.0001^†^	0.9 ± 2.1, 0.7 (−0.6, 2.2)	<0.0001^†^
Percentage time in glucose range, %									
<54 mg/dL	0.65 ± 1.30, 0.28 (0.00, 0.77)	0.23 ± 0.28, 0.18 (0.06, 0.29)	0.29 ± 0.36, 0.18 (0.08, 0.38)	−0.42 ± 1.21, −0.11 (−0.44, 0.04)	<0.0001^††^	−0.36 ± 1.21, −0.03 (−0.41, 0.07)	0.0008^††^	0.06 ± 0.21, 0.03 (−0.02, 0.11)	0.0001^††^
<70 mg/dL	2.93 ± 3.19, 2.05 (0.60, 4.10)	1.34 ± 1.11, 1.09 (0.46, 1.80)	1.52 ± 1.36, 1.23 (0.62, 1.93)	−1.59 ± 2.64, −0.89 (−2.27, 0.02)	<0.0001^††^	−1.41 ± 2.70, −0.86 (−2.11, 0.17)	<0.0001^††^	0.18 ± 0.76, 0.09 (−0.12, 0.36)	0.0070^††^
>180 mg/dL	33.4 ± 17.1, 30.1 (20.7, 46.8)	24.9 ± 11.1, 23.2 (17.0, 31.4)	25.6 ± 11.7, 24.9 (17.6, 32.8)	−8.6 ± 12.2, −7.1 (−16.1, 0.6)	<0.0001^††^	−7.8 ± 12.0, −6.9 (−14.1, 1.1)	<0.0001^††^	0.7 ± 5.6, 0.7 (−1.6, 3.5)	0.1141^††^
≥250 mg/dL	10.5 ± 10.5, 6.7 (2.7, 16.2)	5.8 ± 5.3, 3.9 (2.3, 7.8)	6.5 ± 5.9, 5.0 (2.3, 9.0)	−4.7 ± 7.9, −2.5 (−8.1, 0.1)	<0.0001^††^	−4.0 ± 7.8, −1.6 (−6.6, 0.6)	<0.0001^††^	0.7 ± 3.1, 0.4 (−0.5, 1.7)	0.0036^††^
≥300 mg/dL	3.8 ± 5.5, 1.8 (0.3, 5.1)	1.7 ± 2.4, 0.9 (0.3, 2.2)	2.1 ± 3.1, 1.2 (0.3, 2.8)	−2.1 ± 4.4, −0.6 (−3.0, 0.1)	<0.0001^††^	−1.7 ± 4.2, −0.3 (−2.4, 0.4)	0.0001^††^	0.4 ± 1.8, 0.1 (−0.1, 0.6)	0.0012^††^

Data are mean ± SD, median (IQR). To convert the values for glucose to millimoles per liter, multiply by 0.05551.

^a^ *p*-value determined using ^†^unadjusted two-sided paired *t*-tests or ^††^two-sided Wilcoxon signed rank test.

^b^ Coefficient of variation of sensor glucose is SD divided by the mean.

IQR, interquartile range; SD, standard deviation; ST, standard therapy.

Secondary outcomes demonstrated improvement in time spent in hypoglycemia for adolescents and adults, with a decrease in time <70 mg/dL of median (IQR) 0.86% (−2.11, 0.17), from 2.05% (0.60, 4.10) with standard therapy to 1.23% (0.62, 1.93) during the extension phase (*P* < 0.0001) (Fig. 1C and Table 3). Adolescents and adults also had a median (IQR) reduction of 0.03% (−0.41, 0.07) in time <54 mg/dL (*P* = 0.0008) from standard therapy to extension. In children, time <54 mg/dL increased by median (IQR) 0.07% (−0.06, 0.26) (*P* = 0.0049) from standard therapy to extension, and time <70 mg/dL remained unchanged (Fig. 1C and Table 2).

**FIG. 3.**
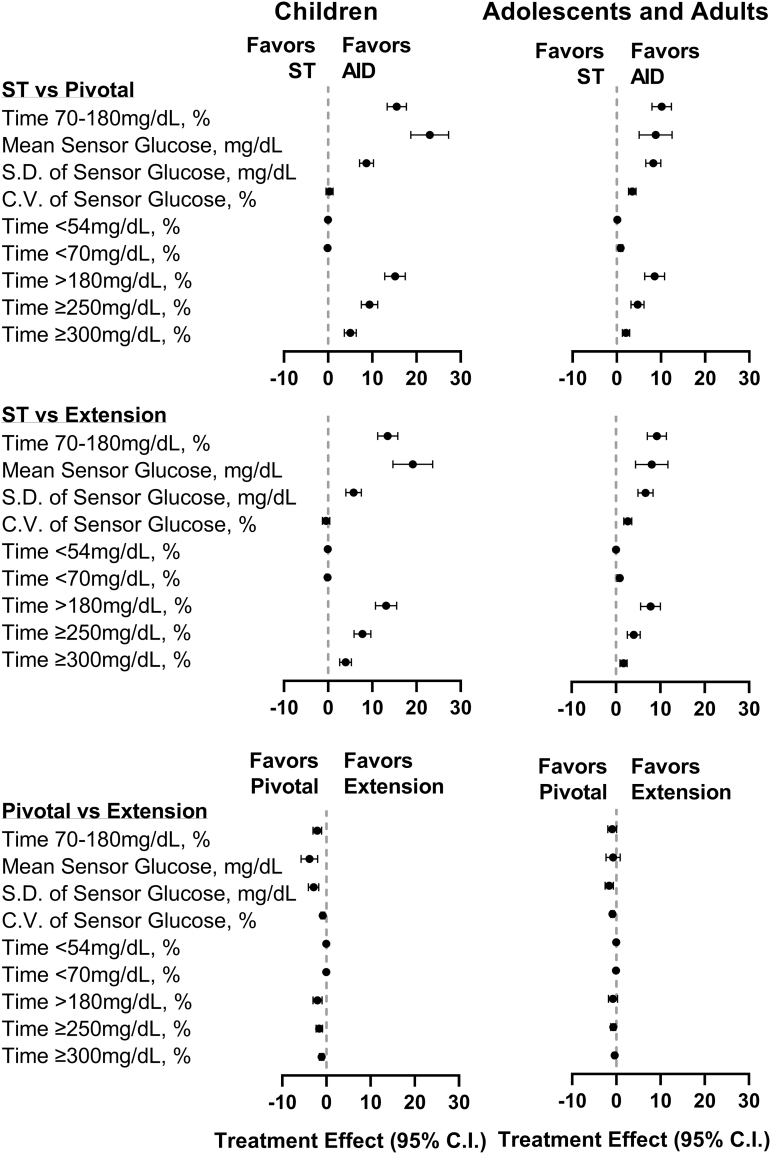
Treatment effect on CGM metrics. Forest plot of CGM outcomes in children (age 6–13.9 years) (left) and adolescents and adults (age 14–70 years) (right) during the ST phase, pivotal AID phase, and extension AID phase. Treatment effect for each metric was calculated such that a positive treatment effect indicated an improvement. Time <54 and <70 mg/dL are shown as median with the 95% CI. All other data are shown as mean with the 95% CI. AID, automated insulin delivery; CGM, continuous glucose monitor; CI, confidence interval; CV, coefficient of variation; SD, standard deviation; ST, standard therapy.

Time spent in hyperglycemia was reduced for both age groups: time >180 mg/dL decreased by mean ± SD 13.1% ± 12.7% from 45.5% ± 16.6% with standard therapy to 32.3% ± 9.5% (*P* < 0.0001) during the extension for children and by 7.8% ± 12.0% from 33.4% ± 17.1% with standard therapy to 25.6% ± 11.7% (*P* < 0.0001) during the extension for adolescents and adults. Improvements in these secondary outcomes were largely maintained from the original 3-month pivotal study, with minor increases in time <54 and <70 mg/dL in adolescents and adults and minor increases in time <54 and >180 mg/dL in children from the pivotal to extension phase (Fig. 3 and Tables 2 and 3).

Glycemic outcomes stratified by adolescent and young adult (ages 14 to <26 years) and adult (ages ≥26 years) age groups are presented in Supplementary Table S5. Both age groups saw improvements in TIR from standard therapy to the pivotal phase, which were largely maintained during the extension, with adults settling higher at 74.1% ± 12.1% compared to 69.7% ± 8.0% for adolescents and young adults (*P* = 0.0180). TIR for both age groups measured in 3-month intervals up to 24 months is displayed in Supplementary Figure S2. Percentage of time <70 mg/dL and HbA1c were similar for both age groups with standard therapy and during the pivotal and extension phases (all *P* > 0.05).

### Safety outcomes

The observed incidence rates of severe hypoglycemia and diabetic ketoacidosis during the extension phase for all participants were 2.04 and 0.24 events per 100 person-years, respectively (Supplementary Table S6). There were seven severe hypoglycemia events: all occurring in children and unrelated to the study device, either following user-initiated boluses (*n* = 2), exercise (*n* = 4), or alcohol consumption while in manual mode (*n* = 1). There was one case of diabetic ketoacidosis in a child, due to a suspected infusion set failure. Additional details on all adverse events recorded during the extension in each age group and overall are available in Supplementary Table S6.

### System use

During the extension phase, children spent median (IQR) 96.1% (92.7, 97.6) and adolescents and adults spent 96.3% (92.3, 97.5) of time in Automated Mode, with steady use over time when analyzed in 3-month intervals (Fig. 1D). There were 0.31 device deficiencies per person-month of extension system use: 71.8% related to the Pod, 16.3% related to the app/handheld device, 7.9% related to the glucose transmitter, and 4.0% related to the glucose sensor.

### Insulin and body weight

Total daily insulin requirements increased in children, from 0.85 ± 0.24 units/kg (U/kg) with standard therapy to 0.92 ± 0.25 U/kg (*P* < 0.0001 from baseline) during the pivotal phase and to 1.04 ± 0.28 U/kg (*P* < 0.0001 from baseline) during the extension, and remained unchanged in adolescents and adults (Supplementary Tables S7 and S8). Children delivered a mean ± SD of 7.1 ± 2.7 boluses/day with standard therapy, which was maintained at 6.7 ± 2.0 boluses/day during the pivotal phase (*P* = 0.1460 from baseline) and decreased to 5.9 ± 2.0 boluses/day during the extension (*P* < 0.0001 from baseline) (Supplementary Table S7). The percentage of total daily insulin from user-initiated boluses in children decreased from 56.9% ± 10.1% with standard therapy to 49.4% ± 6.8% during the pivotal phase (*P* < 0.0001 from baseline) and further decreased to 46.5% ± 7.7% during the extension (*P* < 0.0001 from baseline).

The number of daily boluses in adolescents and adults increased from 6.0 ± 2.7 boluses/day with standard therapy to 7.1 ± 2.9 boluses/day during the pivotal phase (*P* < 0.0001 from baseline) and returned to 6.4 ± 2.6 boluses/day during the extension (*P* = 0.1256 from baseline) (Supplementary Table S8). The percentage of total daily insulin from user-initiated boluses during standard therapy for adolescents and adults was unchanged during the pivotal phase (*P* = 0.6336 from baseline) and decreased slightly from 49.5% ± 8.4% during the pivotal phase to 48.1% ± 8.9% during the extension (*P* = 0.0011).

BMI z-score increased slightly in children from 0.42 ± 0.79 at baseline to 0.52 ± 0.83 (*P* = 0.0248) at 15 months, indicating weight gain compared to normal growth. Participants in the children cohort were aged 7.3 to 15.2 years at the time of final BMI measurement. There was no change in BMI in adolescents and adults.

## Discussion

This multicenter, single-arm, outpatient study provides the longest prospective follow-up of a cohort with established T1D initiating an AID system published to date, with an additional 1 to 1.5 years of data beyond what is available from existing studies. The results demonstrate that the safety and improved glycemic outcomes reported in the pivotal 3-month Omnipod 5 trial persisted for up to 2 years of home use.

HbA1c remained stable from the initial improvements seen in the pivotal trial (from 7.7% ± 0.9% [61 ± 9.8 mmol/mol] to 7.0% ± 0.6% [53 ± 6.6 mmol/mol] for children and from 7.2% ± 0.9% [55 ± 9.8 mmol/mol] to 6.8% ± 0.7% [51 ± 7.7 mmol/mol] for adolescents and adults)^11^ to the end of the extension phase, settling at 7.2% ± 0.7% (55 ± 7.7 mmol/mol) for children and 6.9% ± 0.6% (52 ± 6.6 mmol/mol) for adolescents and adults at 15 months (further HbA1c measurements were not taken).

Likewise, improvement in TIR was maintained with the continued use of Omnipod 5. TIR increased from 52.5% ± 15.6% to 68.0% ± 8.1% for children and from 64.7% ± 16.6% to 73.9% ± 11.0% for adolescents and adults during the pivotal trial^11^ and remained steady at 65.9% ± 8.9% for children and 72.9% ± 11.3% for adolescents and adults in the extension. A significant decrease in time below range (<70 mg/dL) was sustained for the extension in adolescents and adults, while this measure remained low and within recommended targets for children.

Novel to this study is its long duration, which provides the ability not only to assess the durability of the glycemic improvements first reported in the pivotal trial but also to examine users' long-term adoption of the system. Indeed, over 90% of participants entering the extension phase elected to continue in the study until the system became commercially available, indicating high interest to continue use of the system for their or their children's diabetes management. In addition, 86.2% of participants used the system for more than 21 months, providing the longest reported prospective follow-up data to date on extended use of an AID system in a cohort with established T1D.

This study duration is notable as long-term data are key to assessing the durability of the glycemic outcomes achieved following AID use beyond the short-term results collected for regulatory purposes, particularly in pediatric age groups where results can be impacted by normal childhood development. Our results collectively demonstrate that the initial glycemic improvements first reported in the Omnipod 5 pivotal trial are largely sustained over time across both age groups. Notably, these results were achieved while maintaining a high percentage of time in Automated Mode (median 96.1% in children and 96.3% in adolescents and adults), a challenge that has been reported for some AID systems.^13–16^

Our results are consistent with published findings of other commercially available AID systems that have demonstrated glycemic benefit over up to 1 year of use.^17–22^ In a shorter trial of an AID system in children aged 6–13 years (*N* = 78) there was a TIR improvement of 14% during the initial ∼4-month study, which remained stable for up to ∼6 months of total use.^17^ Petrovski et al. reported use of another AID system for 12 months in a small cohort aged 7–18 years (*N* = 30) with improvement in TIR of 27% and a decrease in HbA1c of 1.1%.^18^ Importantly, these participants all transitioned from multiple daily injections and had substantially higher HbA1c at baseline than the present study. An evaluation of the next generation of the AID system from Petrovski et al. in a larger cohort that included children and adults (*N* = 135) showed an improvement in TIR of 10% after 12 months of use.^20^

Although differences in study design limit direct comparison of outcomes (baseline characteristics, sample size, study duration, etc.), the results reported here indicate that the present system compares favorably with other commercially available AID systems and provide new evidence as to the durability of outcomes after up to 2 years of AID. Taken together, these results suggest that the initial short-term glycemic outcomes resulting from AID use across systems are largely maintained over time, provided that system use is continued.

A key feature of the present study is its ability to provide data surrounding rare adverse events, which are challenging to detect and may be underestimated in shorter and smaller studies. The pivotal and extension phases combined provided 4839 person-months of system use (50.7% of this in children), which is over six times that of the original pivotal study; and for the children group alone more than four times that of an extension trial of another AID system in children.^17^ With this long duration of study, event rates for severe hypoglycemia and diabetic ketoacidosis remained quite low at 2.04 and 0.24 events per 100 person-years, which is lower than the observed incidence rates first reported in the pivotal trial (4.8 and 1.2 events per 100 person-years for severe hypoglycemia and diabetic ketoacidosis, respectively^11^).

Further, these rates are substantially lower than the respective rates of 25.2 severe hypoglycemia events and 10.8 diabetic ketoacidosis events per 100 person-years calculated from data reported in the United States T1D Exchange Registry.^3,4^ Studies have recently demonstrated the clinical benefit of early adoption of diabetes technology from disease onset in people with T1D^22–24^ and consensus guidance states that early AID initiation from diagnosis may improve long-term outcomes and reduce health disparities.^25^ These results add confidence in the long-term safety of the system and support the need for future studies investigating the benefits of initiating Omnipod 5 earlier than 6 months after diagnosis. Moreover, these long-term safety outcomes, coupled with maintaining glycemic benefits for up to 2 years, are particularly reassuring for children and adolescents whose insulin needs change throughout normal growth and pubertal development.

Important strengths of this study are the long duration for which participants were followed while using the Omnipod 5 System, providing some of the longest follow-up data for an AID system published to date, and fewer study visits in the extension phase than in the initial pivotal trial, which is more reflective of real-life conditions and may lessen the “study effect” on the reported glycemic outcomes. Furthermore, temporary improvements resulting from the novelty factor of initiating a new system may have diminished, particularly after up to 2 years. Finally, the large sample size of the current study led to a robust dataset, and the broad age range of participants and the multicenter nature of the study also led to enrollment of a diverse pediatric and adult population from across the United States.

A limitation of this work is the lack of a control group due to the single-arm nature of the study design, thus we cannot be certain that the improvement in glycemic outcomes solely resulted from AID use. In addition, the open-ended nature of the study resulted in varying duration of use across participants; however, a difference of a few months is not expected to impact the results given that most participants were on the system for over 21 months.

In conclusion, the safety and improved glycemic outcomes observed as part of the 3-month pivotal trial using the Omnipod 5 System in participants with T1D were maintained for up to 2 years of use, indicating the potential long-term benefit of Omnipod 5 use.

## Conclusions

AID systems have shown great promise in helping people with diabetes achieve their glycemic goals. The Omnipod 5 AID System was shown to be safe and effective in children, adolescents, and adults initiating the system in a 3-month outpatient pivotal trial; however, data on the long-term benefit of Omnipod 5 use is limited. To examine the durability of the pivotal trial results, this study assessed glycemic outcomes in the participants who continued use of the Omnipod 5 AID System in an extension phase for up to 2 years of use. The results demonstrated that the safety and improved glycemic outcomes first observed in the pivotal trial are durable for up to 2 years of at-home use of the system in children, adolescents, and adults with T1D.

These findings not only provide the longest prospective follow-up data on a group of people with established T1D initiating an AID system to date, but also support the long-term benefit of Omnipod 5 use across a diverse range of people with T1D.

## Supplementary Material

Supplemental data
