# Supplementary material for: Two Years with a Tubeless Automated Insulin Delivery System: A Single-Arm Multicenter Trial in Children, Adolescents, and Adults with Type 1 Diabetes
Source: Diabetes Technol Ther. 2024 Jan 5;26(1):11–23. doi: 10.1089/dia.2023.0364 (PMC10794844; doi:10.1089/dia.2023.0364)
Supplement: Supplemental data [file Suppl_Material.docx]

# SUPPLEMENTARY APPENDIX

This appendix has been provided by the authors to give readers additional information about their work.

Supplement to: Criego, Carlson, et al. Two Years with a Tubeless AID System: A Single-Arm Multicenter Trial in Children, Adolescents, and Adults with Type 1 Diabetes

Contents

[SUPPLEMENTARY APPENDIX 1](#_Toc145316088)

[Supplementary Table S1. Eligibility and Exclusion Criteria 2](#_Toc145316089)

[Supplementary Table S2. Schedule of participant visits 4](#_Toc145316090)

[Supplementary Table S3. HbA1c Results over time for participants electing to participate in the extension phase* 7](#_Toc145316091)

[Supplementary Table S4. Number of participants meeting consensus targets (1) for glycemic control during the standard therapy phase and the extension phase for both children and adults 9](#_Toc145316092)

[Supplementary Table S5. Glycemic outcomes stratified by adolescent and young adult (ages 14 to <26 years) and adult (ages ≥26 years) age groups 10](#_Toc145316093)

[Supplementary Table S6. Safety outcomes during extension phase^*^ 11](#_Toc145316094)

[Supplementary Table S7. Insulin requirements during the standard therapy phase and extension phase for children (ages 6-13.9 years) 12](#_Toc145316095)

[Supplementary Table S8. Insulin requirements during the standard therapy phase and extension phase for adolescents and adults (ages 14-70 years) 13](#_Toc145316096)

[Supplementary Figure S1. Subgroup analysis of HbA1c in 3-month intervals stratified by baseline HbA1c 14](#_Toc145316097)

[Supplementary Figure 2. Subgroup analysis of TIR in 3-month intervals stratified by age group 15](#_Toc145316098)

[REFERENCES 16](#_Toc145316099)

## Supplementary Table S1. Eligibility and Exclusion Criteria

| *Inclusion Criteria*  Subjects must meet all of the following criteria to be included in the study:   1. Age at time of consent/assent 6-70 years 2. Subjects aged < 18 years must be living with parent/legal guardian 3. Diagnosed with type 1 diabetes for at least 6 months. Diagnosis is based on investigator’s clinical judgment 4. Deemed appropriate for pump therapy per investigator’s assessment taking into account previous history of severe hypoglycemic and hyperglycemic events, and other comorbidities 5. Investigator has confidence that the subject can successfully operate all study devices and is capable of adhering to the protocol 6. Willing to use only the following types of insulin during the study: Humalog, Novolog, Admelog or Apidra during the study 7. Must be willing to travel to and participate in meal and exercise challenges during 5-days of the hybrid closed-loop phase 8. Willing to wear the system continuously throughout the study 9. A1C <10% at screening visit 10. Must be willing to use the Dexcom App on the Omnipod Horizon™ PDM as the sole source of Dexcom data (with the exception of the Dexcom Follow App) during the hybrid closed-loop phase 11. Subjects scoring ≥ 4 on the Clarke Questionnaire must agree to have an overnight companion, defined as someone who resides in the same home or building as the study subject and who can be available overnight 12. Able to read and speak English fluently 13. Willing and able to sign the Informed Consent Form (ICF) and/or has a parent/guardian willing and able to sign the ICF. Assent will be obtained from pediatric and adolescent subjects aged < 18 years per State requirements. |
| --- |
| *Exclusion Criteria*  Subjects who meet any of the following criteria will be excluded from the study:   1. A medical condition, which in the opinion of the investigator, would put the subject at an unacceptable safety risk 2. History of severe hypoglycemia in the past 6 months 3. History of DKA in the past 6 months, unrelated to an intercurrent illness, infusion set failure or initial diagnosis 4. Diagnosed with sickle cell disease 5. Diagnosed with hemophilia or any other bleeding disorders 6. Plans to receive blood transfusion over the course of the study 7. Currently diagnosed with anorexia nervosa or bulimia 8. Acute or chronic kidney disease (e.g. estimated GFR < 45) or currently on hemodialysis 9. History of adrenal insufficiency 10. Has taken oral or injectable steroids within the past 8-weeks or plans to take oral or injectable steroids during the course of the study 11. Unable to tolerate adhesive tape or has any unresolved skin condition in the area of sensor or pump placement 12. Plans to use insulin other than U-100 insulin intended for use in the study device during the course of the study 13. Use of non-insulin anti-diabetic medication other than metformin (e.g. GLP1 agonist, SGLT2 inhibitor, DPP-4 inhibitor, pramlintide) 14. Current or known history of coronary artery disease that is not stable with medical management, including unstable angina, or angina that prevents moderate exercise despite medical management, or a history of myocardial infarction, percutaneous coronary intervention, or coronary artery bypass grafting within the previous 12-months. 15. For subjects >50 years old or with diabetes duration >20 years, abnormal electrocardiogram consistent with increased risk of arrhythmia, ischemia, or prolonged QT_c_ interval (> 450 ms) 16. Thyroid Stimulating Hormone (TSH) is outside of normal range with clinical signs of hypothyroidism or hyperthyroidism 17. Pregnant or lactating, or is a woman of childbearing potential and not on acceptable form of birth control (acceptable includes abstinence, condoms, oral/injectable contraceptives, IUD or implant) 18. Participation in another clinical study using an investigational drug or device other than the Omnipod Horizon™ Automated Glucose Control System within the preceding 30-days or intends to participate during the study period 19. Unable to follow clinical protocol for the duration of the study or is otherwise deemed unacceptable to participate in the study per the investigator’s clinical judgment |

## Supplementary Table S2. Schedule of participant visits

| **Assessment Schedule** | **Automated Insulin Delivery Phase (AID) - Extension** | | | | | | | | | | |  |  |
| --- | --- | --- | --- | --- | --- | --- | --- | --- | --- | --- | --- | --- | --- |
|  | **Phase 3** | | | | | | | | | | |  |  |
| Visit Number | 14 | 15 | 16 | 17 | 18 | 19 | 20 | 21 | 22 | 23  or Study Exit | Extension Through Commercialization^o^ | UV | EW |
| Study Day/Visit Window | 120 ± 5d | 150 ± 5d | 180 ± 5d | 210 ± 5d | 240 ± 5d | 270 ± 5d | 315 ± 5d | 360 ± 5d | 405 ± 5d | 450 ± 5d | + 60d ± 5d  (or sooner if Study Exit) |  |  |
| Telephone (T) or Office (O) Visit | T/O^f^ | T/O^f^ | T/O^f^ | T/O^f^ | T/O^f^ | T/O^f^ | T/O^f^ | T/O^f^ | T/O^f^ | T/O^f^ | T/O^f^ | T/O^f^ | O |
| **Laboratory Assessments** | | | | | | | | | | | | | |
| A1C |  |  | X |  |  | X |  | X |  | X |  |  | X |
| Pregnancy Test |  |  | X^f^ |  |  | X^f^ |  |  |  |  |  |  |  |
| **Clinical Assessments** | | | | | | | | | | | | | |
| Informed Consent |  |  |  |  |  | X^m^ |  |  |  | X^m^ |  |  |  |
| Concomitant Medications | X | X | X | X | X | X | X | X | X | X | X | X | X |
| Height |  |  |  |  |  |  |  |  |  | X |  |  | X |
| Weight |  |  |  |  |  |  |  |  |  | X |  |  | X |
| Adverse Events | X | X | X | X | X | X | X | X | X | X | X | X | X |
| **Study Devices** | | | | | | | | | | | | | |
| Return Horizon System |  |  |  |  |  |  |  |  |  | X^p^ | X^p^ |  | X |
| Complaints/Device Deficiencies | X^h^ | X^h^ | X^h^ | X^h^ | X^h^ | X^h^ | X^h^ | X^h^ | X^h^ | X^h^ | X^h^ | X^h^ | X^h^ |
| Device Uploads (BG/Ketone Meter) | X^f^ | X^f^ | X^f^ | X^f^ | X^f^ | X^f^ | X^f^ | X^f^ | X^f^ | X^f^ | X^f^ |  | X |
| Data Review | X^n^ | X^n^ | X^n^ | X^n^ | X^n^ | X^n^ | X^n^ | X^n^ | X^n^ | X^n^ | X^n^ | X^n^ | X^n^ |

Abbreviations: S=Screening; ST1=Standard Therapy Day One; HCL=Hybrid Closed-loop; EW=Early Withdrawal; QC=Quality Control Testing; UV=Unscheduled Visit

| ^a^Early withdrawal visit will only be conducted for any subjects that started but did not complete the full study to include standard therapy and the Automated Insulin Delivery Phase (AID) . |
| --- |
| ^b^Unscheduled visits will serve as extra study visits, if needed. For unscheduled visits pertaining to a study pause and recommencement, sites should follow the assessments as defined in section 9.10. |
| ^c^Challenges can occur during any consecutive 5-days during Phase 2 of the Automated Insulin Delivery Phase (AID). A follow up telephone visit will occur the following day after the conclusion of the challenge period. |
| ^d^Electrocardiogram required for subjects >50 years old or with diabetes duration >20 years |
| ^e^Subjects extending into the pivotal study from the prepivotal study will initiate their participation at Visit 5 of pivotal Phase 2. |
| ^f^Visits identified as "T/O" can either be conducted in person at the clinical site or over the telephone. Visits identified as "O" can only be conducted in person at the clinical site. Vital signs, device uploads/data review from the BG and Ketone meter, and pregnancy tests are not required at any visit conducted via telephone. |
| ^g^Prepivotal subjects extending into pivotal will be required to reconsent. If original prepivotal screening is within 45 days prior to the start of Phase 2 in the pivotal study, subjects will not require rescreening. All subjects must reconsent prior to commencing the extension phase (Phase 3) on or before Visit 13. |
| ^h^Documentation only applicable if there are changes from previous assessment |
| ^i^Study device training for the CGM, blood glucose and ketone meters |
| ^j^Study device training for the Omnipod Horizon™ System |
| ^k^Subjects deemed exempt from wearing the study CGM for 14-days will be eligible to immediately commence the hybrid closed-loop phase at Visit 4 and may skip Visit 3/ST2 (in which case, Visit 1, Visit 2/ST1 and Visit 4 may all occur on the same day) |
| ^l^In the event of overlapping visit windows, no visits occurring during Phase 2 are to occur on the same date, except for challenge visit days. It is acceptable for a challenge visit to occur on the same date as a Phase 2 visit |
| ^m^Subjects extending their participation into the extension phase are to be consented for the first 6-month extension, the second 6-month extension, and the extension through commercial availability. Consent for each interval must occur on or any time before commencing that study interval (e.g. consent for first 6-months of Phase 3 must occur on or before Visit 13, consent for second 6-months of Phase 3 must occur on or before Visit 19, consent for visits beyond Visit 23 until commercial availability must occur on or before Visit 23).  ^n^Data review for Horizon Automated Mode to occur at all visits  ^o^ Each Extension Through Commercialization (ETC) visit begins with Visit 24 and each additional visit will be numbered in consecutive order ending with the Study Exit visit.  ^p^Only perform assessment if this is the final study visit |

## Supplementary Table S3. HbA1c Results over time for participants electing to participate in the extension phase*

| **Parameter** | **Baseline** | **3 Months**  **(Pivotal End)** | **6 Months** | **9 Months** | **12 Months** | **15 Months**^†^ |
| --- | --- | --- | --- | --- | --- | --- |
| **Children** |  |  |  |  |  |  |
| N with HbA1c available | 110 | 108 | 108 | 110 | 108 | 109 |
| HbA1c (%, mmol/mol) | 7.7 ± 0.9,  61 ± 9.8 | 7.0 ± 0.6,  53 ± 6.6 | 6.9 ± 0.6,  52 ± 6.6 | 7.0 ± 0.7,  53 ± 7.7 | 7.0 ± 0.6,  53 ± 6.6 | 7.2 ± 0.7,  55 ± 7.7 |
| HbA1c (%, mmol/mol) change from baseline | --- | -0.7 ± 0.6,  -7.7 ± 6.6 | -0.8 ± 0.7,  -8.7 ± 7.7 | -0.7 ± 0.7,  -7.7 ± 7.7 | -0.6 ± 0.7,  -6.6 ± 7.7 | -0.5 ± 0.7,  -5.5 ± 7.7 |
| P-value from baseline | --- | <0.0001^1^ | <0.0001^1^ | <0.0001^1^ | <0.0001^2^ | <0.0001^1^ |
| % with HbA1c <7% | 25 (23%) | 57 (53%) | 58 (54%) | 60 (55%) | 47 (44%) | 40 (37%) |
| **Adolescents and Adults** |  |  |  |  |  |  |
| N with HbA1c available | 114 | 114 | 107 | 108 | 99 | 103 |
| HbA1c (%, mmol/mol) | 7.2 ± 0.9,  55 ± 9.8 | 6.8 ± 0.7,  51 ± 7.7 | 6.7 ± 0.6,  50 ± 6.6 | 6.7 ± 0.7,  50 ± 7.7 | 6.8 ± 0.6,  51 ± 6.6 | 6.9 ± 0.6,  52 ± 6.6 |
| HbA1c (%, mmol/mol) change from baseline | --- | -0.4 ± 0.6,  -4.4 ± 6.6 | -0.5 ± 0.6,  -5.5 ± 6.6 | -0.5 ± 0.6,  -5.5 ± 6.6 | -0.4 ± 0.7,  -4.4 ± 7.7 | -0.3 ± 0.6,  -3.3 ± 6.6 |
| P-value from baseline | --- | <0.0001^2^ | <0.0001^2^ | <0.0001^1^ | <0.0001^2^ | <0.0001^1^ |
| % with HbA1c <7% | 48 (42%) | 74 (65%) | 78 (73%) | 72 (67%) | 57 (58%) | 61 (59%) |

^*^Data are mean ± SD, unless otherwise indicated.

^†^HbA1c was not measured post-15-months of total system use.

^1^Unadjusted two-sided *p*-value for paired t-test.

^2^Two-sided Wilcoxon signed rank test.

## Supplementary Table S4. Number of participants meeting consensus targets (1) for glycemic control during the standard therapy phase and the extension phase for both children and adults

|  | **Children (6 to 13.9 years)**  **(n=110)** | | **Adults (14 to 70 years)**  **(n=114)** | |
| --- | --- | --- | --- | --- |
| Number of participants meeting target, n (%) | **Baseline**^†^ **or Standard Therapy Phase** | **Follow-up**^†^ **or Extension Phase** | **Baseline**^†^ **or Standard Therapy Phase** | **Follow-up**^†^ **or Extension Phase** |
| HbA1c* <7.0% (<53 mmol/mol) | 25 (23%) | 40 (37%) | 48 (42%) | 61 (59%) |
| HbA1c* <7.5% (<58 mmol/mol) | 54 (49%) | 72 (66%) | 77 (68%) | 77 (75%) |
| Time in range 70-180mg/dL >60% | 33 (30%) | 82 (75%) | 68 (60%) | 99 (87%) |
| Time in range 70-180mg/dL >70% | 17 (15%) | 33 (30%) | 47 (41%) | 73 (64%) |
| Time <70mg/dL <4% | 94 (85%) | 102 (93%) | 82 (72%) | 107 (94%) |
| Composite – Time in range >60% and time <70mg/dL <4% | 24 (22%) | 74 (67%) | 46 (40%) | 92 (81%) |
| Composite – Time in range >70% and time <70mg/dL <4% | 12 (11%) | 27 (25%) | 33 (29%) | 67 (59%) |

*Baseline HbA1c values were available for 110 children and 114 adults. Final HbA1c values were available for 109 children and 103 adults at 15 total months of AID use.

^†^Baseline and follow-up data were used for the primary effectiveness outcomes of HbA1c, the remaining outcomes are described for the standard therapy phase and the extension phase.

## Supplementary Table S5. Glycemic outcomes stratified by adolescent and young adult (ages 14 to <26 years) and adult (ages ≥26 years) age groups

| **Age Group** | **14 to <26 years (n=31)** | | | **≥26 years (n=83)** | | | **14 to <26 years vs. ≥26 years** | | |
| --- | --- | --- | --- | --- | --- | --- | --- | --- | --- |
| **Parameter** | **ST Phase** | **Pivotal Study** | **Extension Study** | **ST Phase** | **Pivotal Study** | **Extension Study** | **p-value (ST)** | **p-value (Pivotal)** | **p-value (Extension)** |
| **Percentage time 70-180mg/dL, %** | 57.6 ± 14.6  55.5 [48.1, 70.6] | 70.9 ± 9.4  73.7 [64.1, 78.3] | 69.7 ± 8.0  70.2 [65.0, 75.8] | 65.9 ± 16.6  68.3 [51.9, 78.6] | 74.9 ± 11.2  75.8 [69.1, 83.3] | 74.1 ± 12.1  75.2 [67.2, 83.1] | **0.0132^2^** | 0.0677^2^ | **0.0180^2^** |
| **Percentage time <70mg/dL, %** | 2.9 ± 2.8  2.7 [0.5, 4.4] | 1.6 ± 1.2  1.4 [0.8, 1.8] | 1.7 ± 1.2  1.5 [1.0, 2.3] | 3.0 ± 3.3  2.0 [0.6, 4.0] | 1.2 ± 1.1  1.0 [0.4, 1.8] | 1.5 ± 1.4  1.0 [0.5, 1.8] | 0.7623^2^ | 0.0763^2^ | 0.0512^2^ |
| **HbA1c, %** | 7.4 ± 0.8  7.4 [6.6, 8.1] | 6.8 ± 0.7  6.7 [6.4, 7.3] | 7.0 ± 0.5  6.9 [6.5, 7.5] | 7.1 ± 0.9  7.1 [6.6, 7.6] | 6.8 ± 0.7  6.7 [6.4, 7.2] | 6.9 ± 0.7  6.9 [6.3, 7.4] | 0.1829^1^ | 0.8354^2^ | 0.4428^1^ |

Data are mean ± SD, median [IQR]. IQR denotes interquartile range. *p*-value determined using ^1^unpaired t-tests or ^2^Mann-Whitney tests.

## Supplementary Table S6. Safety outcomes during extension phase^*^

|  | **Children**  **(6 to 13.9 years)**  **(n=110)** | **Adults**  **(14 to 70 years)**  **(n=114)** | **Total**  **(6 to 70 years)**  **(n=224)** |
| --- | --- | --- | --- |
| **Event Type** |  |  |  |
| **Primary Safety Outcomes**  **(events per 100 person-years)**^†^ |  |  |  |
| Severe hypoglycemia | 3.96 | 0.00 | 2.04 |
| Diabetic ketoacidosis | 0.60 | 0.00 | 0.24 |
| Hypoglycemia, number of events (% of participants)^‡^ | 0 (0.0) | 1 (0.9) | 1 (0.4) |
| Severe Hypoglycemia, number of events (% of participants)^§^ | 7 (6.4) | 0 (0.0) | 7 (3.1) |
| Diabetic Ketoacidosis, number of events (% of participants)^\|\|^ | 1 (0.9) | 0 (0.0) | 1 (0.4) |
| Hyperglycemia, number of events (% of participants)^¶^ | 6 (3.6) | 3 (2.6) | 9 (3.1) |
| Prolonged Hyperglycemia, number of events (% of participants)^#^ | 37 (22.7) | 4 (3.5) | 41 (12.9) |
| Other, number of events (% of participants)^**^ | 41 (30.9) | 26 (19.3) | 67 (25.0) |

^*^Extension phase was calculated from the end of the pivotal clinical trial (initial 3 months of AID use) to the end of the extension phase.

^†^Rates of severe hypoglycemia and diabetic ketoacidosis derived from the United States T1D Exchange were 25.2 and 10.8 per 100 person-years, respectively (2, 3).

^‡^Hypoglycemia resulting in a serious adverse event but otherwise not meeting the definition of severe hypoglycemia

^§^Severe hypoglycemia requiring the assistance of another person due to altered consciousness, and requiring another person to actively administer carbohydrate, glucagon, or other resuscitative actions

^||^Hyperglycemia with the presence of polyuria, polydipsia, nausea or vomiting, serum ketones >1.5mmol/L or large/moderate urine ketones, either arterial blood pH <7.30, venous pH <7.24, or serum bicarbonate <15, and treatment provided in a health care facility.

^¶^Hyperglycemia requiring evaluation, treatment or guidance from intervention site, or hyperglycemia resulting in a serious adverse event but otherwise not meeting the definition of DKA or prolonged hyperglycemia.

^#^Meter blood glucose measuring ≥300mg/dL and ketones >1.0mmol/L

^**^Other related, but non-glycemic adverse events included infection or irritation at infusion site (6 children, 5 adults). Other events unrelated to the study device included viral illnesses (e.g. COVID-19, oral thrush, pharyngitis), tonsillectomy and adenoidectomy, gastroenteritis, skin abscess, broken limbs, concussion, pneumomediastinum, syncope, pregnancy, suicidal ideation, and suspected heart attack, stroke, or aneurysm. There was one death, following the suspected heart attack, stroke, or aneurysm.

## Supplementary Table S7. Insulin requirements during the standard therapy phase and extension phase for children (ages 6-13.9 years)

| **Insulin Requirement** | **Standard Therapy Phase** | **Pivotal Study** | **Extension Study** | **Change from Standard Therapy to Extension** | ***p*-value*** |
| --- | --- | --- | --- | --- | --- |
|  | **(n=110)** | **(n=110)** | **(n=110)** | **(n=108)** |  |
| Total daily insulin  (U/kg) | 0.85 ± 0.24,  0.82 (0.69, 1.00) | 0.92 ± 0.25,  0.88 (0.75, 1.09) | **(n=108)**  1.04 ± 0.28,  0.99 (0.85, 1.18) | **(n=108)**  0.18 ± 0.24,  0.17 (0.04, 0.34) | <0.0001^2^ |
| Total daily basal insulin (U/kg) | 0.36 ± 0.12,  0.35 (0.28, 0.42) | 0.47 ± 0.15,  0.44 (0.37, 0.56) | **(n=108)**  0.55 ± 0.16,  0.55 (0.45, 0.64) | **(n=108)**  0.19 ± 0.15,  0.17 (0.10, 0.30) | <0.0001^1^ |
| Total daily bolus insulin (U/kg) | 0.49 ± 0.18,  0.46 (0.36, 0.58) | 0.45 ± 0.13,  0.45 (0.36, 0.53) | **(n=108)**  0.48 ± 0.16,  0.46 (0.37, 0.57) | **(n=108)**  -0.01 ± 0.19,  0.01 (-0.10, 0.08) | 0.9733^2^ |
| Total daily insulin  (U) | 34.5 ± 17.6,  29.6 (21.8, 45.0) | 37.5 ± 19.7,  33.4 (23.9, 48.6) | 44.7 ± 21.4,  41.9 (29.2, 57.4) | 10.2 ± 11.5,  7.7 (3.0, 17.5) | <0.0001^2^ |
| Total daily basal insulin (U) | 14.7 ± 8.3,  13.6 (8.6, 18.2) | 19.2 ± 10.6,  16.4 (11.1, 25.3) | 24.0 ± 11.5,  23.2 (15.0, 31.4) | 9.3 ± 7.6,  8.4 (3.5, 15.1) | <0.0001^1^ |
| Total daily bolus insulin (U) | 19.8 ± 11.1,  16.9 (12.0, 27.0) | 18.3 ± 9.6,  16.3 (11.4, 22.5) | 20.8 ± 11.0,  17.9 (13.2, 25.6) | 1.0 ± 8.8,  1.5 (-2.2, 4.6) | 0.0569^2^ |
| Number of boluses (per day) | 7.1 ± 2.7,  6.6 (5.0, 8.0) | 6.7 ± 2.0,  6.3 (5.5, 7.5) | 5.9 ±2.0,  5.8 (4.5, 7.2) | -1.1 ± 2.5,  -0.9 (-2.4, 0.3) | <0.0001^2^ |
| Insulin from user-initiated boluses (%) | 56.9 ± 10.1,  57.8 (51.8, 63.4) | 49.4 ± 6.8,  49.4 (45.1, 53.6) | 46.5 ± 7.7,  45.7 (41.2, 51.0) | -10.4 ± 11.9,  -10.7 (-17.5, -3.3) | <0.0001^1^ |

Data are mean ± SD, median (IQR). IQR denotes interquartile range.

^*^*p*-value determined using unadjusted two-sided paired t-tests, unless otherwise specified.

^1^Unadjusted two-sided p-value for paired t-test.

^2^Two-sided Wilcoxon signed rank test.

## Supplementary Table S8. Insulin requirements during the standard therapy phase and extension phase for adolescents and adults (ages 14-70 years)

| **Insulin Requirement** | **Standard Therapy Phase** | **Pivotal Study** | **Extension Study** | **Change from Standard Therapy to Extension** | ***p*-value*** |
| --- | --- | --- | --- | --- | --- |
|  | **(n=114)** | **(n=114)** | **(n=114)** | **(n=114)** |  |
| Total daily insulin  (U/kg) | 0.61 ± 0.22,  0.58 (0.48, 0.71) | 0.60 ± 0.21,  0.53 (0.45, 0.74) | **(n=113)**  0.60 ± 0.19,  0.54 (0.47, 0.72) | **(n=113)**  -0.01 ± 0.11,  0.01 (-0.06, 0.05) | 0.9444^2^ |
| Total daily basal insulin (U/kg) | 0.30 ± 0.10,  0.31 (0.24, 0.36) | 0.30 ± 0.11,  0.29 (0.22, 0.38) | **(n=113)**  0.31 ± 0.10,  0.30 (0.23, 0.38) | **(n=113)**  0.01 ± 0.09,  0.01 (-0.04, 0.06) | 0.2897^1^ |
| Total daily bolus insulin (U/kg) | 0.31 ± 0.16,  0.28 (0.19, 0.39) | 0.30 ± 0.12,  0.27 (0.21, 0.35) | **(n=113)**  0.29 ± 0.12,  0.26 (0.21, 0.33) | **(n=113)**  -0.01 ± 0.09,  0.01 (-0.08, 0.04) | 0.3870^2^ |
| Total daily insulin  (U) | 48.3 ± 21.0,  45.1 (35.7, 54.9) | 46.8 ± 18.0,  45.4 (33.1, 56.5) | 47.8 ± 18.8,  45.0 (33.5, 57.6) | -0.5 ± 9.9,  0.4 (-4.7, 3.5) | 0.9698^2^ |
| Total daily basal insulin (U) | 24.0 ± 10.4,  24.1 (16.7, 30.0) | 23.7 ± 9.9,  23.4 (16.0, 29.6) | 24.7 ± 10.1,  23.9 (17.1, 30.4) | 0.7 ± 7.1,  0.6 (-3.4, 5.7) | 0.3008^1^ |
| Total daily bolus insulin (U) | 24.3 ± 13.6,  21.9 (14.4, 30.1) | 23.1 ± 9.7,  21.2 (16.4, 27.9) | 23.1 ± 10.7,  20.9 (15.8, 28.3) | -1.2 ± 7.2,  0.0 (-4.9, 3.1) | 0.3048^2^ |
| Number of boluses (per day) | 6.0 ± 2.7,  5.4 (4.1, 7.4) | 7.1 ± 2.9,  6.5 (5.0, 8.8) | 6.4 ± 2.6,  5.8 (4.6, 8.4) | 0.4 ± 2.2,  0.1 (-0.8, 1.4) | 0.1256^2^ |
| Insulin from user-initiated boluses (%) | 49.0 ± 12.2,  50.3 (39.9, 57.0) | 49.5 ± 8.4,  50.2 (44.4, 54.1) | 48.1 ± 8.9,  48.6 (42.5, 53.3) | -0.9 ± 11.0,  -1.1 (-8.9, 7.1) | 0.3883^1^ |

Data are mean ± SD, median (IQR). IQR denotes interquartile range.

^*^*p*-value determined using unadjusted two-sided paired t-tests, unless otherwise specified.

^1^Unadjusted two-sided p-value for paired t-test.

^2^Two-sided Wilcoxon signed rank test.

## Supplementary Figure S1. Subgroup analysis of HbA1c in 3-month intervals stratified by baseline HbA1c

HbA1c for children (age 6-13.9 years) (left) and adolescents and adults (age 14-70 years) (right) in 3-month intervals stratified by baseline HbA1c <8% (top) and ≥8% (bottom). Dashed line represents the consensus target of HbA1c 7.0%. Error bars show the standard deviation. **p*<0.05, ***p*<0.01, and ****p*<0.001. Abbreviations: AID-automated insulin delivery, ST-standard therapy


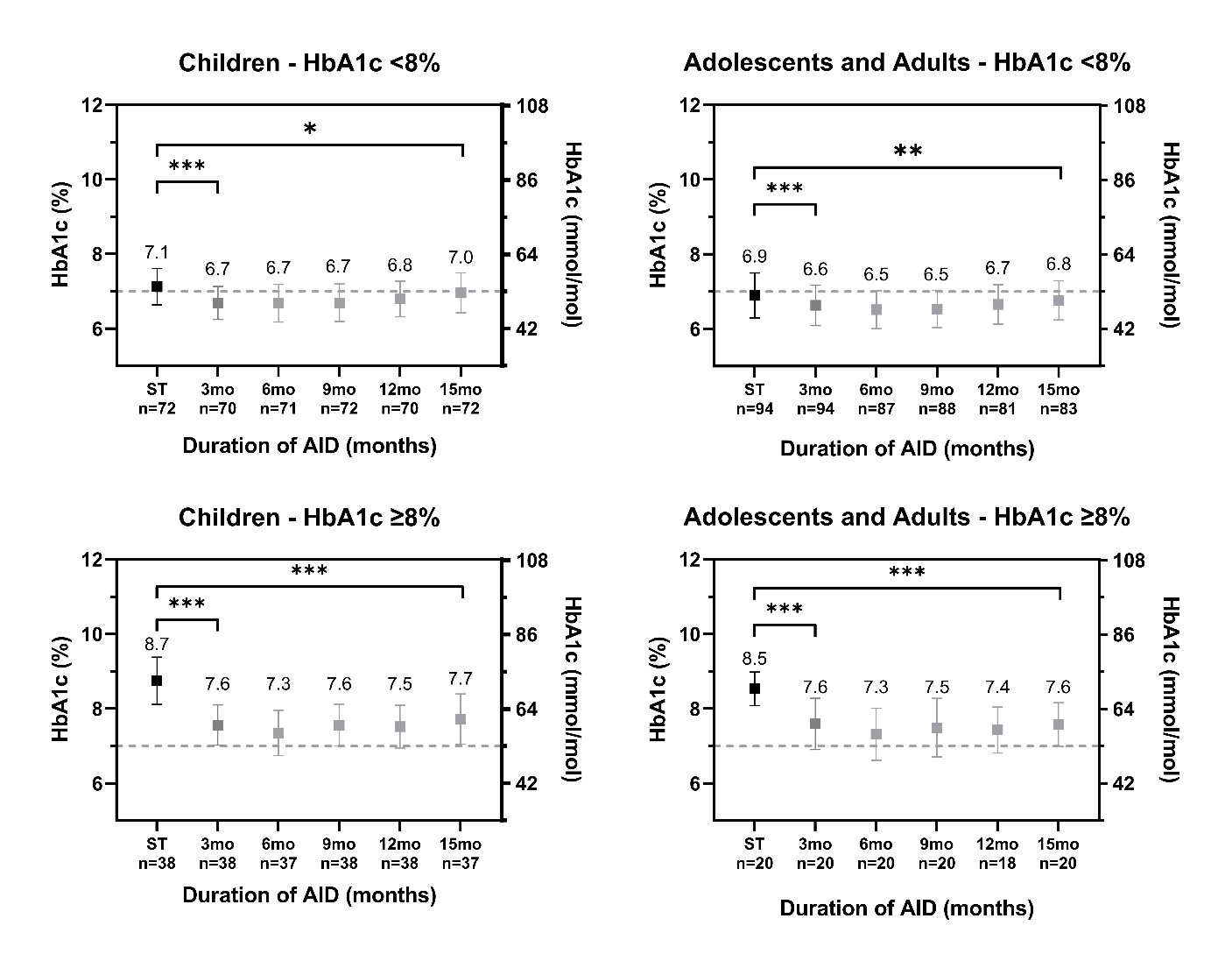


## Supplementary Figure 2. Subgroup analysis of TIR in 3-month intervals stratified by age group

Percentage time in target range (TIR) 70-180mg/dL during the standard therapy phase (ST), pivotal phase, and extension phase for adolescents and young adults (age 14 to <26 years) (left) and adults (age ≥26 years) (right) in 3-month intervals. Box plots represent the median (line) with mean (dots). ****p*<0.001. Abbreviations: AID-automated insulin delivery, ST-standard therapy


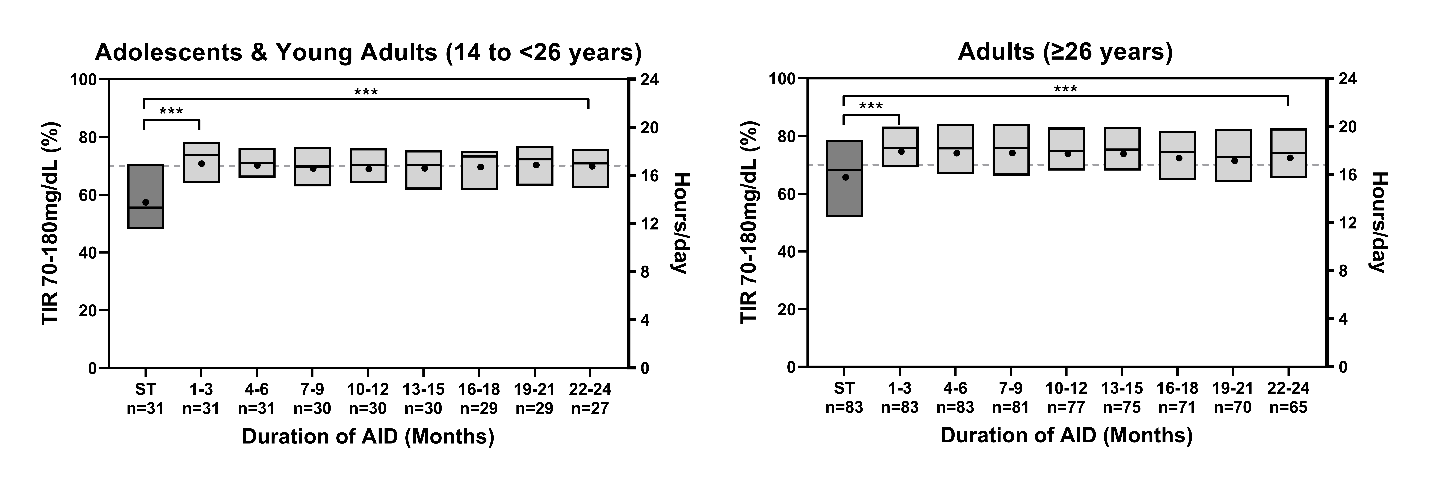


## REFERENCES

1. ElSayed NA, Aleppo G, Aroda VR, et al. 6. Glycemic Targets: Standards of Care in Diabetes—2023. *Diabetes Care.* 2022;46(Supplement_1):S97-S110.
2. Foster NC, Beck RW, Miller KM, et al. State of Type 1 Diabetes Management and Outcomes from the T1D Exchange in 2016-2018. *Diabetes technology & therapeutics.* 2019;21(2):66-72.
3. Miller KM, Foster NC, Beck RW, et al. Current state of type 1 diabetes treatment in the U.S.: updated data from the T1D Exchange clinic registry. *Diabetes Care.* 2015;38(6):971-978.
